# Assessment of Low-Density Force Myography Armband for Classification of Upper Limb Gestures

**DOI:** 10.3390/s23052716

**Published:** 2023-03-01

**Authors:** Mustafa Ur Rehman, Kamran Shah, Izhar Ul Haq, Sajid Iqbal, Mohamed A. Ismail, Fatih Selimefendigil

**Affiliations:** 1 Department of Mechatronics Engineering, University of Engineering and Technology, Peshawar 25000, Pakistan; 2 Department of Mechanical Engineering, King Faisal University, Al-Hofuf 31982, Saudi Arabia; 3 Department of Information Systems, King Faisal University, Al-Hofuf 31982, Saudi Arabia

**Keywords:** accuracy, static protocol, dynamic protocol, force myography (FMG), gestures

## Abstract

Using force myography (FMG) to monitor volumetric changes in limb muscles is a promising and effective alternative for controlling bio-robotic prosthetic devices. In recent years, there has been a focus on developing new methods to improve the performance of FMG technology in the control of bio-robotic devices. This study aimed to design and evaluate a novel low-density FMG (LD-FMG) armband for controlling upper limb prostheses. The study investigated the number of sensors and sampling rate for the newly developed LD-FMG band. The performance of the band was evaluated by detecting nine gestures of the hand, wrist, and forearm at varying elbow and shoulder positions. Six subjects, including both fit and amputated individuals, participated in this study and completed two experimental protocols: static and dynamic. The static protocol measured volumetric changes in forearm muscles at the fixed elbow and shoulder positions. In contrast, the dynamic protocol included continuous motion of the elbow and shoulder joints. The results showed that the number of sensors significantly impacts gesture prediction accuracy, with the best accuracy achieved on the 7-sensor FMG band arrangement. Compared to the number of sensors, the sampling rate had a lower influence on prediction accuracy. Additionally, variations in limb position greatly affect the classification accuracy of gestures. The static protocol shows an accuracy above 90% when considering nine gestures. Among dynamic results, shoulder movement shows the least classification error compared to elbow and elbow–shoulder (ES) movements.

## 1. Introduction

The human upper limb is a dexterous organ through which activities of daily livings (ADLs) can be achieved, such as handshaking, driving, grasping or lifting an object, etc. [1]. It is also important to communicate information or emotions through sign language. Losing the use of one’s upper limb can be devastating as it limits a person’s ability to perform ADLs. Various upper limb prosthetic devices, including body-powered and externally powered prostheses, have been developed to restore functionality. Body-powered prostheses use harness systems and are operated using intact body parts; however, they offer limited functionality and require much power [2]. Externally powered prostheses, particularly myoelectric devices, provide more dexterity, cosmetics, and ease of operation than body-powered prostheses. Various research-based and commercially available prosthetic devices with multiple degrees of freedom (mDOFs) have been developed recently. Among these, the most well-known commercially available upper limb prosthetic hands are Otto Bock’s Michelangelo hand [3], Steeper Group’s Bebionic V3 [4], and Touch Bionic’s i-Limb [5].

Myoelectric prosthetic devices require an interface for converting user intent to myoelectric signals. The popular adopted sensing mechanisms are surface electromyography (sEMG) [6], ultrasound imaging [7], and force myography (FMG) techniques. However, sEMG is a widely used sensing mechanism. FMG is a relatively new technology to sEMG. The other names for FMG are topographic pressure map (TPM) [8] residual kinetic imaging (RKI) [9], and surface pressure mapping (SPM) [10]. This technology is considered a potential replacement for the sEMG technique because of its cost-effectiveness, high signal-to-noise ratio (SNR), and the least effect on muscle fatigue, hair, and sweat [11,12,13]. Force myography is a non-invasive technique that uses pressure sensors to monitor volumetric changes in the musculotendinous complex during gesture contractions. These pressure sensors are either piezoresistive [14], piezoelectric [15], or capacitive [16]. However, resistive-based sensors such as force-sensitive resistors (FSRs) are more popular and widely used in this technique [16]. This technique provides a user-friendly, cost-effective, and unobtrusive method to monitor volumetric muscle changes. Furthermore, FMG signals can be generated using commercially available pressure sensors and do not require complex circuitry for signal processing [17]. In addition, it is not necessary to place FMG sensors on the specific anatomical points of the muscles to acquire adequate myoelectric signals [8]. Considering these factors, many studies are examining the use of FMG to monitor muscle activity, gesture recognition, and detection of functional tasks. With machine learning and pattern recognition, this sensing mechanism has been gaining importance in the last decade to detect limb activities, hand gestures, and grip repetitions.

No consistent standard has been adopted in developing the FMG system regarding sensor quantity selection and sampling rate required for effectively detecting upper limb movements. Some studies [18,19,20] used low-density pressure mapping (three to eight sensors), whereas others developed customized sensor arrays for FMG applications [21,22]. These high-density sensor arrays give more promising results compared to low-density sensor FMG bands. However, they add complexity, weight, cost, and computational time to the system, leading to increased maintenance and manufacturing costs for prostheses. Therefore, low-density FMG bands are utilized to recognize a limited number of gestures. As far as the sampling rate is concerned, it varies among studies from 6 Hz to 1 kHz. The sampling rate of 6 Hz is sufficient for static gesture recognition. However, higher frequencies are recommended for dynamic gestures, including multiple hands, forearm, and elbow movements, or their combination [16].

FMG technology is utilized to detect upper limb activities, including finger forces [23], fine finger movements [24], grip strength [25], hand gestures classification [26], and monitoring wrist and forearm gestures [27]. Most of these studies were performed under static limb conditions. For example, during experimentation, subjects were seated on a comfortable chair with their elbow and shoulder fixed positions. These static conditions provide higher accuracies for gestures classification. However, these conditions are far away from real-life scenarios. Since subjects are supposed to move a limb in space to achieve their ADLs, these variations and orientations in limb position cause a degradation in classification accuracy when the classifier is trained on a static condition and tested on multiple orientations or dynamic movements. Due to these orientation and position changes, different volumetric muscle patterns develop for the same gesture. Therefore, dynamic protocols were developed to train the classifier on multiple arm positions or orientations to effectively recognize gesture patterns in multiple positions and orientations of the limb. Radmond et al. [28] were the first to develop a dynamic protocol for FMG-based techniques. Their study classified a set of eight hand and wrist gestures using static and dynamic protocols. Later, Freigo et al. [29] introduced a dynamic protocol by covering generic humeral and transverse planes.

Similarly, Ahmadizadeh et al. [26] also utilized a dynamic protocol for training their classifier for ten hand, wrist, and forearm gestures. All these studies showed that limb position variation affects gesture classification accuracy. Table 1 shows a literature review of FMG-based studies.

This study investigates the impact of sensor quantity and sampling rate on the performance of a novel low-density force myography (LD-FMG) armband to control upper limb prostheses. The study also examines the effect of limb position variation on the classification accuracy of upper limb gestures, including fine finger movements and hand movements. A dynamic protocol was designed to assess the impact of the elbow, shoulder, and combined elbow–shoulder movements on gestures classification. The results of this study will provide insights into the fundamental parameters of FMG technology and its potential for use in bio-robotic devices.

## 2. Materials and Methods

### 2.1. FMG Sensors

FMG technology, which measures volumetric changes in muscle activity, often uses force-sensitive resistors (FSRs) as the sensing mechanism. Its cost-efficiency, lightweight design, precision, and small size make it suitable for bio-robotic applications [40]. This study used seven commercially available force-sensitive resistors (FSR) from Interlink (model No. 402) to develop the FMG armband. These FSRs have a 12.07 mm^2^ sensing area and a sensitivity range from 0.2 to 20 N [41]. The FSRs were chosen for their cost-effectiveness, light weight, accuracy, and compact size, which make them suitable for bio-robotic applications. However, FSR sensors are flexible and can bend when placed on human skin, leading to uneven pressure distribution [16,42,43]. To address this issue, a stiff mechanical housing was designed for each FSR sensor to protect it from bending and to ensure that muscle pressure is focused solely on the sensitive area of the sensor.

A novel mechanical housing was developed to protect the FSR sensors from bending and to ensure that muscle pressure is focused on the sensitive area of the sensor. The design consists of two halves, with the sensor embedded in the lower housing.

**Figure 1:**
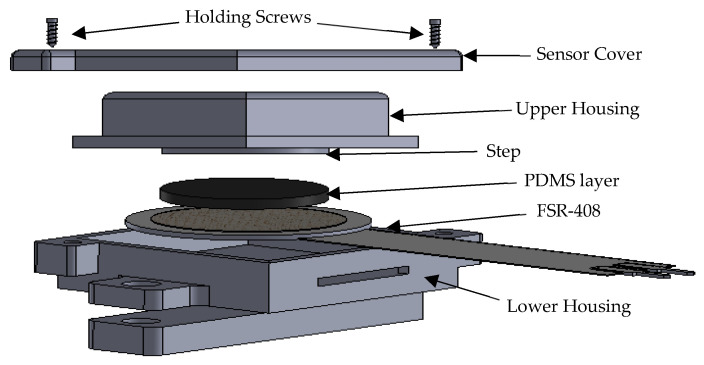
Exploded view of FSR sensor with housing.

The upper housing has a circular step of 12 mm in diameter and a 1.2 mm polydimethylsiloxane (PDMS) layer at the lower end. The upper end is the force-sensing tip that transmits muscle–tendon force to the FSR. The housing is 3D printed using PLA, providing rigidity and being safe for direct skin contact due to its biocompatibility [44]. The new FSR housing developed in this study measures 2.3 cm × 2 cm × 1 cm. It was designed to protect the FSR from bending and to ensure that pressure is applied only to the sensitive area of the sensor. As previously performed in other studies, the FSR sensors were mounted on a Velcro strap and placed evenly around the subject’s forearm [19,27]. The developed FSR sensor’s exploded view is depicted in Figure 1.

### 2.2. Data Acquisition and Transfer Setup

**Figure 2:**
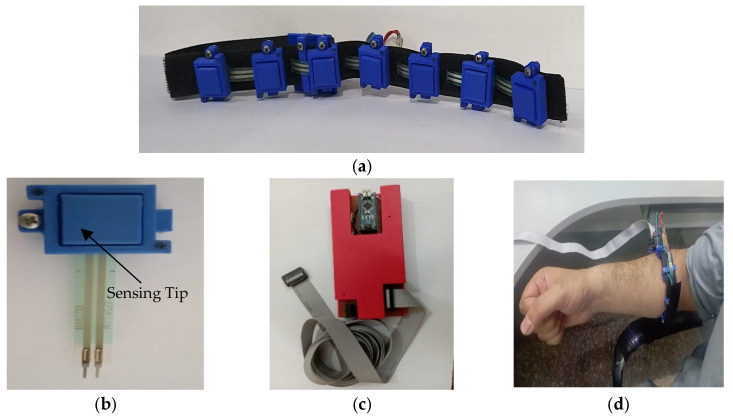
(**a**) FMG armband with 7S sensors, (**b**) 3D printed sensor, (**c**) Data acquisition hardware, and (**d**) LD-FMG band fastened on individual’s forearm.

In this study, a voltage divider circuit, effective in previous research [31,45], was utilized to extract the signals from the FSR sensors. The circuit includes ground resistors (Rg) to adjust the sensitivity of the FSR and determines the relationship between the maximum force before saturation and the maximum output voltage of the FSR. The value of *R_g_* selected in this study was 20 kΩ. The signals from the FSR were quantified using an operational amplifier (op-amp) from an Arduino Nano board, which features a 16 MHz ATmega328 microprocessor and a 10-bit analog-to-digital converter (ADC). The complete hardware setup is shown in Figure 2a–d.

### 2.3. Experimental Setup

**Figure 3:**
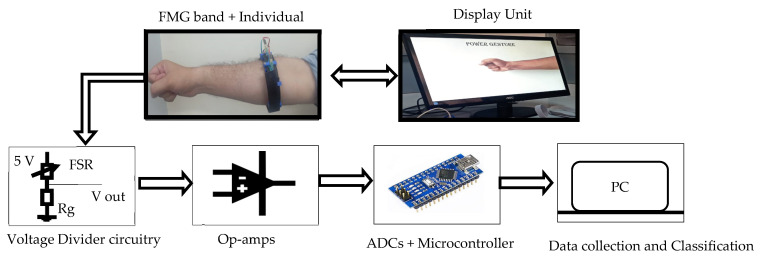
Flow chart of the experimental setup.

An experimental setup consisting of the FMG band, data collection, and classification setup was developed. Figure 3 shows the flowchart of the experimental setup from data recording to data classification. The FMG band’s FMG sensors perceive volumetric changes in muscles and FMG signals are digitized using the Arduino Nano board.

### 2.4. Subjects

This experimental study recruited six volunteer adult participants, three of whom were fit, and three had trans-radial amputations. The participants ranged from 28 to 45 years, and all were physically active and had no skin-related issues. The study’s developed FMG band and experimental procedures were thoroughly explained to the participants. The research was approved by the ethical committee of the University of Engineering and Technology Peshawar, Pakistan (UET Peshawar), and all participants signed an informed consent form before the experimental procedure began.

### 2.5. Experimental Protocol

**Figure 4:**
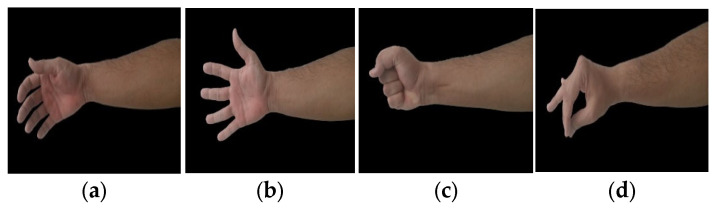

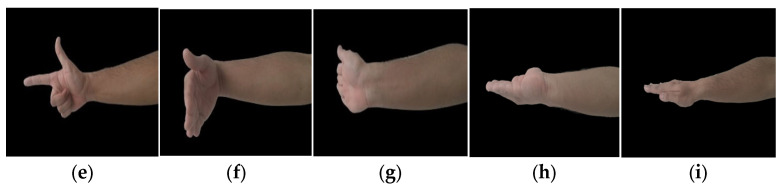
Upper limb gestures: (**a**) Relax, (**b**) fingers extended, (**c**) power, (**d**) tripod, (**e**) finger point, (**f**) wrist flexion, (**g**) wrist extension, (**h**) forearm supination, and (**i**) forearm Pronation.

Two protocols were developed for data collection: one for static and the other for dynamic limb positions. Data were collected for nine upper extremity gestures, including five major hand gestures, mostly performed to accomplish activities of daily living (ADLs) according to [29,34,46], such as relax, fingers extended, power, tripod, and finger point. In addition, two wrist movements, such as wrist flexion and wrist extension [47], and two forearm movements, such as forearm supination and forearm pronation [48], are shown in Figure 4. For both protocols, the FMG band was positioned on the upper portion of the forearm. The participants could record data while standing or sitting on an armless chair. Participants were also asked for feedback on the tightness and comfort of the band during the experiment. The gestures to be performed and their names were displayed on the host device. Participants were instructed to perform the gestures with consistent force. Data recording for both protocols was conducted on the same day for each participant.

#### 2.5.1. Static Limb Position Protocol

**Figure 5:**
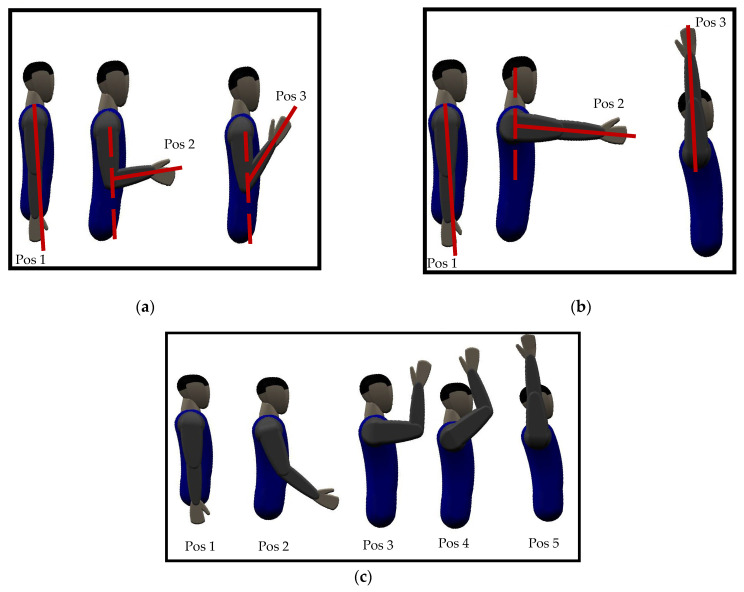
Dynamic motions of upper limb (**a**) elbow, (**b**) shoulder, and (**c**) ES movement.

During static limb position, the elbow and shoulder positions were fixed, and data for the hand, wrist, and forearm gestures, as mentioned earlier, were recorded. Subjects were asked to flex their elbow at position 2 and their shoulder at position 1 (shown in Figure 5). This pose was selected for static protocol because it is the most adopted elbow and shoulder position during ADLs such as handshaking, reaching, and grasping objects, etc. Data collection for gestures from different sets of sensors and sampling rates followed the sequence shown in Table 2. Each gesture was displayed for 5 s on the host device, and subjects were asked to elicit that gesture contraction. Three seconds of rest phase was given between gestures to avoid muscle fatigue. Data were recorded in two sessions. In each session, sequence steps (as shown in Table 2) were repeated three times for data collection. The sequence of gestures remained the same throughout the experiment to minimize participants’ confusion. It was made sure to perform both sessions for a single participant on the same day, and a 20 min break was given between sessions.

##### Effect of the Number of Sensors

The number of sensors selected in this study to recognize all nine gestures was 3, 5, and 7 sets of sensors on the FMG band. The 3-sensor FMG armband is named ‘3S’; similarly, the 5- and 7-sensor FMG armbands are termed ‘5S’ and ‘7S’, respectively. These terminologies will be used throughout the paper to describe LD-FMG armbands with the associated number of sensors. Since the developed armband is based on a low-density pressure mapping technique, the selected number of sensors ranges from three to seven. This selection of the number of sensors is similar to the minimum number of sensors (three to eight) supposed to be sufficient for effectively perceiving user intent in pattern recognition-based studies [20,25]. The other reason for this selection of sensor quantity is that each sensor contributes to the stiffness of the armband. Therefore, increasing the number of sensors may compromise the band’s stiffness and make it difficult to mount on thinner forearms.

##### Effect of Sampling Rate

The data for all nine gestures were recorded at sampling frequencies of 5 Hz, 10 Hz, and 20 Hz, since the frequency of human arm motion is less than 4.5 Hz [49]. Furthermore, based on Nyquist criteria [50] (the minimum sampling frequency is twice the frequency of a moving body), a 10 Hz sampling rate was mainly adopted in previous studies [11,20,26] for data recording of static gestures. Therefore, in this study, data recording was performed at 5 Hz (almost equal to human hand motion), 10 Hz (based on Nyquist criteria), and 20 Hz sampling frequencies to determine the effect of sampling rates on static hand gesture recognition.

#### 2.5.2. Dynamic Limb Position Protocol

Data for the gestures mentioned above were recorded by varying elbow, shoulder, and their combined movements to investigate the limb position effect. The movements were performed in humeral planes since most ADLs are covered in this plane [34,51]. For elbow movements, subjects were instructed to maintain the gesture while continuously moving their elbow from position 1 to position 3 (from extended to flexed position), as shown in Figure 5a, while not moving shoulder position. Similarly, data for gestures were collected for shoulder movements (from position 1 to position 3) while maintaining a fixed elbow position. For combined elbow and shoulder movements (we termed it ‘ES’), subjects were asked to simultaneously move their elbow and shoulder following positions 1-2-3-4-5, as shown in Figure 5c. For each elbow, shoulder, and ES movement, subjects were asked to maintain the gesture and gradually move their upper limb from the starting to the final position in each movement (as shown in Figure 5) for 10 s. If the subject reached the final position before 10 s, they were asked to follow movement in the reverse direction, i.e., the final-to-start position. Data were recorded three times for each elbow, shoulder, and ES movement, and a break of 10 min was given to subjects between movements.

### 2.6. Data Collection

A single trial from the static protocol at a 20 Hz sampling frequency consists of 100 samples for each of the nine gestures (5 s at 20 Hz). Similarly, a dynamic protocol of elbow, shoulder, and ES movement consists of 200 samples (10 s at 20 Hz) for a gesture in a single trial. The data was normalized from 0 to 1 and analyzed using the Statistics and Machine learning Toolbox of MATLAB on a Dell corei3 laptop (Processor: 2.4 GHz, Ram: 8 GB DDR3, Operating system: Windows 10). The classification performance of the data sets acquired from both protocols was evaluated using the support vector machines (SVM) algorithm. The SVM algorithm was chosen as it is the most tested and adopted classifier for bio-signal classification [16,52].

In addition, a k-fold cross-validation scheme (K = 5) was utilized for gesture classification. The mean classification accuracy from cross-validation is presented in this paper.

## 3. Results

### 3.1. Classification Performance of the Static Protocol

#### 3.1.1. Effect of the Number of Sensors on Classification Accuracy

Overall, the classification accuracy across all subjects was highest on the 7S FMG band, followed by the 5S and 3S bands. The average accuracies predicted for the 3S, 5S, and 7S FMG bands were 62.1 ± 6.5%, 81 ± 4.2%, and 91.6 ± 3.7%, respectively.

**Figure 6:**
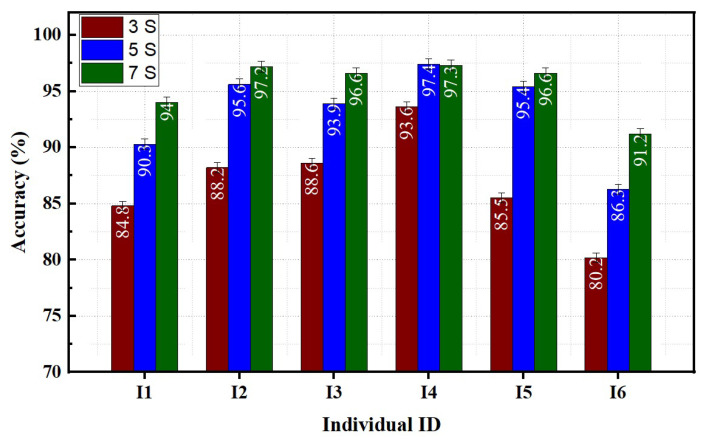
Effect of sensor’s quantity on individual performance.

The effect of the number of sensors on individual participants’ performances across three trials is illustrated in Figure 6. All three sets of sensors demonstrate average classification accuracies greater than 80%. The average accuracy increases as the number of sensors increases. All individuals achieved the maximum average accuracy on the 7S FMG band. Among all participants, participant I4 showed the highest classification accuracy across all sensor sets.

#### 3.1.2. Effect of Sampling Rates on Classification Accuracy

**Figure 7:**
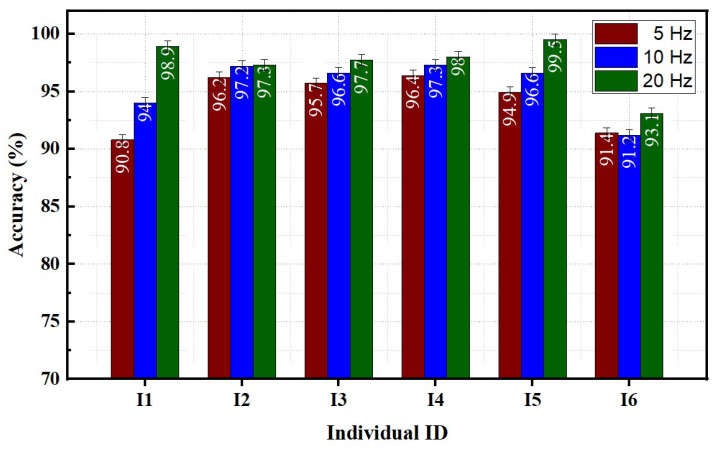
Effect of sampling rates on individual performance.

The classification accuracy tends to increase as the sampling rate increases. The highest average classification accuracy of 95.6 ± 1.6% was achieved at a sampling rate of 20 Hz. In contrast, the lowest accuracy of 86.1 ± 2.9% was obtained at a rate of 5 Hz. Figure 7 illustrates the effect of the data sampling rate on the classification performance of individual participants using the 7S FMG band. The effect of the sampling rate was more pronounced in individuals I1 and I5 compared to others. An average accuracy difference of around 8% was found between the 5 Hz and 20 Hz sampling rate data for individual I1. For individual I5, an increase of 4.5% in accuracy was observed between data acquired at 5 Hz and 20 Hz sampling rates. The sampling frequency did not significantly impact the prediction of gestures for individuals I2, I3, I4, and I6.

### 3.2. Classification Performance of the Dynamic Protocol

**Figure 8:**
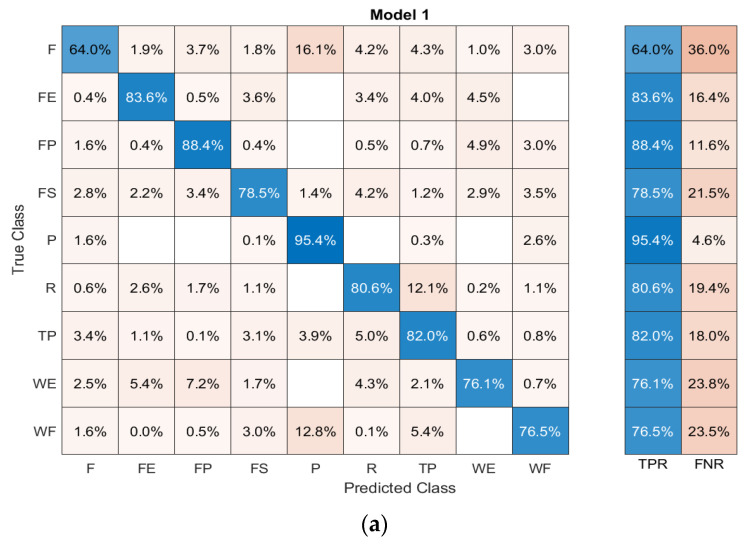

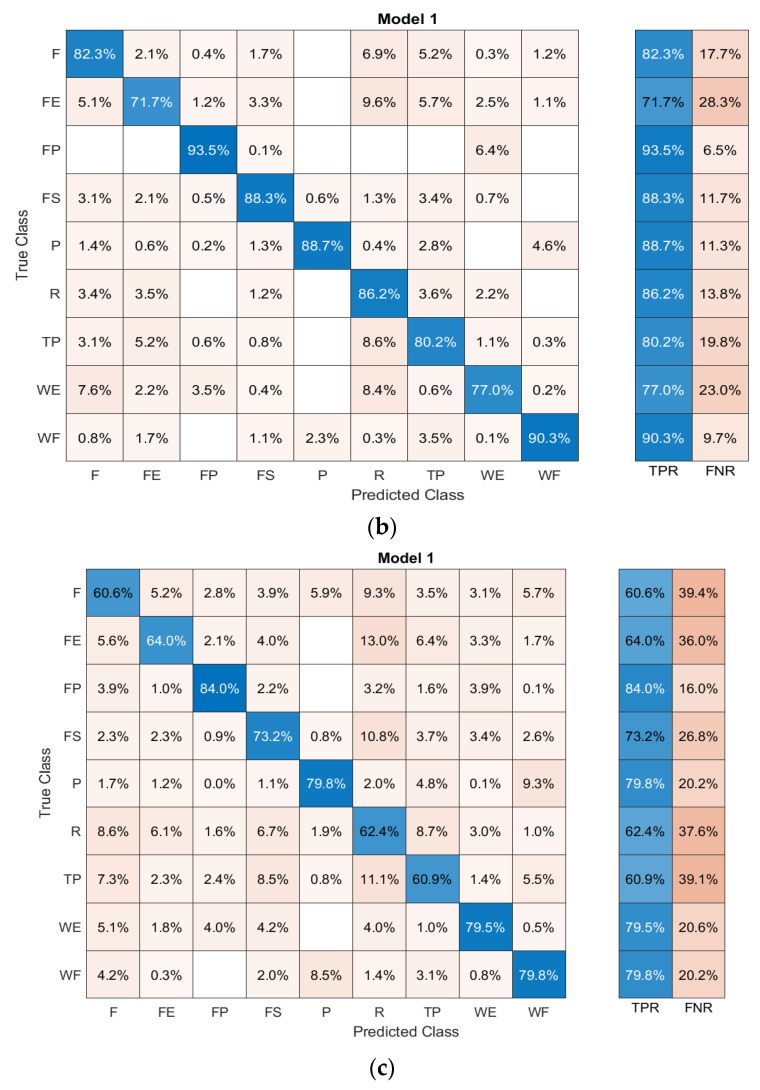
Confusion matrices of (**a**) elbow, (**b**) shoulder, and (**c**) ES movements; F: finger point, FE: finger extension, FP: forearm pronation, FS: forearm supination, P: power gesture, R: relax, TP: tripod, WE: wrist extension, and WF: wrist flexion.

Figure 8a–c displays the confusion matrices from dynamic datasets during the execution of elbow, shoulder, and ES movements. Each confusion matrix illustrates the true and predicted classes of gestures represented by rows and columns. The diagonal entries in the matrix represent the average accuracy of correctly classified classes (true positive rates, TPR), and the average error for misclassified classes (false negative rates, FNR) is shown in other entries. The results indicate that variations in limb position affect the average classification accuracy of gestures. The dynamic movement of the elbow achieves an average accuracy of up to 80.6 ± 5.2% for classifying nine gestures. Shoulder and ES movements have average accuracies of 84.3 ± 3.7% and 71.3 ± 6.4%, respectively.

### 3.3. Effect of Limb Position Variation on Hand Gestures

**Figure 9:**
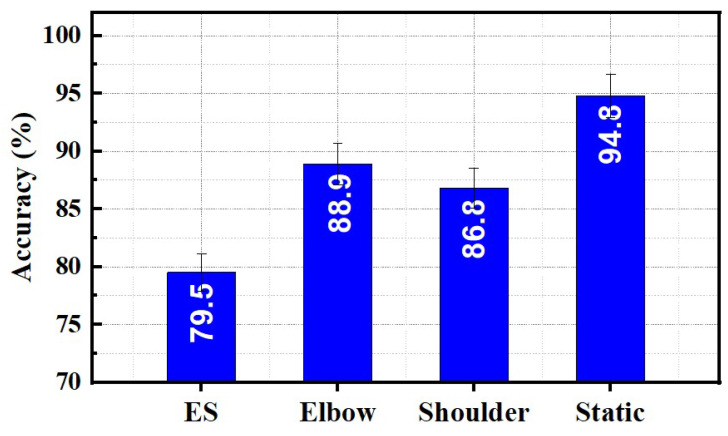
Comparison of static and dynamic protocol on hand gestures classification performance.

When recognizing five hand gestures, the static limb position achieves a maximum average classification accuracy of 94.8%. However, variations in limb position led to an increase in classification error. As seen in Figure 9, classification accuracies were obtained under different limb movement conditions. The maximum classification error was around 21% for ES movement, and the minimum was around 12% for shoulder movement. Elbow movement demonstrates an average classification accuracy of 86.8% for hand gestures.

### 3.4. Effect of Limb Position Variations on Wrist and Forearm Gestures

**Figure 10:**
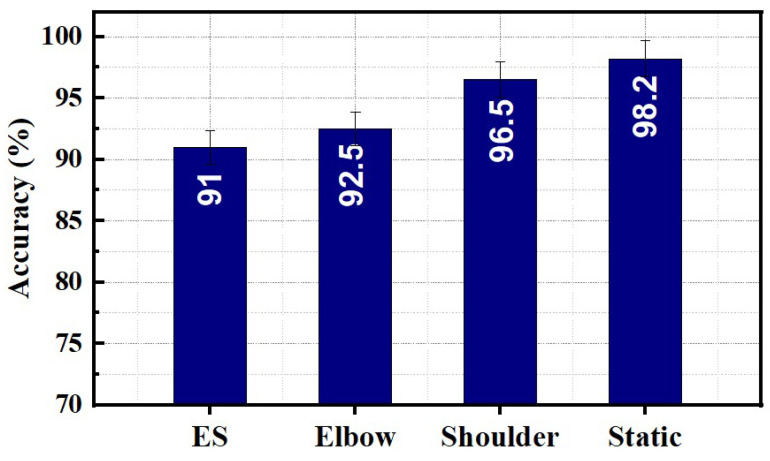
Comparison of static and dynamic protocol on wrist and forearm gestures classification performance.

Figure 10 compares the average classification accuracies of wrist and forearm gestures for static and dynamic limb movements. An average accuracy above 90% was observed for static and dynamic protocols. Static limb position demonstrates maximum average accuracy of 98.2%. Elbow, shoulder, and ES movements showed average accuracies of 91.2%, 92.9%, and 96.9%, respectively.

## 4. Discussion

An effective gesture detection technique is essential for an efficient control system of bio-robotic assistive devices. This study presented an LD-FMG band with seven newly developed FSR sensors. Static and dynamic protocols were designed to investigate upper limb gestures.

The effect of sensor quantity and sampling rates on the performance of the newly developed FMG band was investigated. As presented in Section 3.1, the number of sensors for perceiving user intent significantly impacts gesture recognition. The performance of the armband rises with the increasing number of sensors. Average accuracy increases by around 30% by increasing the number of sensors from 3S to 5S. Comparing 5S and 7S, an improvement in prediction accuracy of approximately 10% is observed. This relationship between the number of sensors and gesture recognition is also evident in previous studies. Lei et al. [38] demonstrated that the accuracy of hand gesture recognition increases from 77.92 % to 99.18% by increasing the channel number from 2 to 8. Ahmadizadeh et al. [26] demonstrated that accuracy for predicting 3 to 10 classes of upper limb motions increases from 74% to 95% for the same set of sensors. Similarly, studies [11,27] showed improvement in accuracy with an increasing number of channels for data collection.

The sampling rate at which data is recorded also affects the prediction accuracy of the gestures. However, the influence of the sampling rate on prediction accuracy is lower than the number of sensors. An increase of around 5% in prediction accuracy is achieved by increasing the sampling rate from 5 Hz to 10 Hz. Beyond 10 Hz, an additional improvement of around 4% in prediction accuracy is observed. The influence of the sampling rate from 5 Hz to 10 Hz may be due to the wrist and forearm movements in the selected gestures.

The results of the study indicate that the performance of the static protocol was significantly better than the dynamic protocol in predicting classes of upper limb gestures. This outcome was expected as FMG monitored the forearm muscles. Due to variations in elbow positions, there were volumetric changes in the forearm muscles. The human forearm consists of intrinsic, extrinsic, and brachioradialis muscles. Intrinsic muscles mainly cause pronation and supination in the forearm, extrinsic muscles move finger joints, and the brachioradialis is responsible for elbow joint flexion and extension [53].

The brachioradialis is located on the lateral side of the forearm and extends from the upper arm to the wrist. Volumetric changes in the forearm muscles due to the flexion and extension of the elbow joint result in distinct myoelectric signals for the same gesture at different limb positions. This finding is consistent with previous studies [28,34], which also reported similar observations. By using an array of sensors, it was found that the position of the upper limb has an impact on the accuracy of gesture classification. Furthermore, it was determined that FMG, which detects changes in the volume of forearm muscles, is more effective at detecting variations in the elbow joint than other methods, such as sEMG. Xiao et al. [27] found that FMG is more effective than sEMG in predicting elbow movements, with an 18% higher accuracy. This is due to the minimal change in the electrical activity of the forearm muscles when the elbow is moved.

In comparison, shoulder movements have less impact on upper limb gesture prediction accuracy. The flexion and extension of shoulder movements are caused by the deltoid muscle, which has minimal disruption on the forearm muscles [54]. Furthermore, the dynamic movement of the shoulder has been found to have a 5% higher classification accuracy in predicting nine gestures than elbow movements. Additionally, the dynamic movement of the shoulder demonstrates a 5% higher classification accuracy in predicting nine gestures than elbow movements. In addition, the accuracy is even further increased, up to 18%, for ES movement. Fine finger movements (hand gestures) and hand movements (wrist and forearm gestures) were also found to be less affected by shoulder movements, indicating that shoulder movements have less influence on the upper limbs regardless of the type of gesture.

One more key finding of this study is that the accuracy of gesture prediction also depends on the relationship between the number of sensors and gestures. The study found that the higher the ratio of sensors to gestures, the higher the prediction accuracy. Conversely, the lower the ratio, the lower the accuracy. For example, this study found that wrist and forearm gestures (four gestures) were predicted more accurately than nine. An improvement of approximately 6% in accuracy was observed. This trend of improvement in accuracy with an increasing ratio between the number of gestures and sensors for data collection is consistent with previous literature [11,26,38,55].

The current study has a limitation in that it only examines the dynamic protocol in the humeral plane. Therefore, it is necessary to study further the impact of limb movements in the sagittal and transverse planes. Furthermore, these planes are also crucial in performing activities of daily living, so they may likely effect gesture classification accuracy. Therefore, investigating these planes will aid in developing a more realistic protocol for training algorithms for practical usage. Integration of developed FMG sensors in the prosthetic socket of the upper limb is also essential to make it effective for the end-user. New integration techniques were developed that compact the size of embedded sensors and make them more comfortable for direct interaction with skin [56,57]. Additionally, it would be beneficial to investigate other AI algorithms, including deep learning and various signal feature extraction techniques, such as time-based, frequency-based, and time–frequency-based, in the armband.

## 5. Conclusions

There is a substantial body of research on different techniques developed for FMG-based prosthetic control; however, most studies focus on ideal conditions with limited variation in limb positions. This study, however, aims to develop a novel LD-FMG band for recognizing upper limb gestures, and its contributions are:

- The study results indicate that the number of sensors used in the FMG band significantly impacts its performance, with the 7S FMG band showing higher classification accuracy than the 5S and 3S FMG bands.
- Compared to the number of sensors used, the sampling rate has less of an impact on the performance of the FMG band. The study also determined that a sampling rate of 10 Hz or above is sufficient for recognizing upper limb movements.
- The developed LD-FMG band demonstrates good discrimination between different gestures in static protocols compared to dynamic protocols.
- Among variations in limb positions, the shoulder joint had the least effect on the prediction accuracy of the gestures. However, it was also observed that limb positions affected fine finger movements (hand gestures) and hand movements (wrist and forearm movements).

## Figures and Tables

**Table 1:** Literature review of FMG-based studies.

Ref. No.	Year	Sensor’s Quantity	Sampling Rate (Hz)	Sensor Housing	Protocol Type
[24]	2012	32	100	-	Static
[23]	2014	10	10	No	Static
[20,29]	2014	8	10	No	Static
[30]	2016	8	30	No	Dynamic
[21]	2016	126	20	Socket	Static and Dynamic
[19]	2016	10	10	Yes	Static
[31]	2017	6	10	No	Dynamic
[32]	2017	10	196	Yes	Dynamic
[33]	2017	10	16	Yes	Static
[11]	2017	16	10	No	Static
[26]	2017	16	10	Socket	Dynamic
[34]	2017	80	10	Socket	Static and Dynamic
[35]	2018	8	10	No	Static
[36]	2019	8	1k	No	Static
[18]	2019	3	25	Yes	Static
[37]	2019	8	100	-	Dynamic
[38]	2021	16	1–1k	No	Static
[39]	2022	16	10–50	No	Dynamic

**Table 2:** Data collection sequence.

Session	Sequence Steps	No. of Sensors	Sampling Rate
1	1	3	10
	2	5	10
	3	7	10
2	1	7	5
	2	7	20

## Data Availability

Data available on request due to restrictions.
